# Ulceration of the Uterus

**Published:** 1874-06

**Authors:** Evory Kennedy


*THE OBSTETRICAL JOURNAL—January,* 1874:

ULCERATION OF THE UTERUS—*By Dr. Evory Kennedy.*—It seems a simple matter to introduce a speculum, detect an ulcer accompanied with more or less metritis or congestion of the uterus; to apply caustics, scarify, and administer tonics—and no doubt a certain proportion of the cases presenting themselves may be cured or benefited by this routine treatment—but what of the cases that baffle us?

When we find an eminent Viennese physician (Pollak) thus generalize upon these ulcerations, in cases of chronic metritis and mucous discharges from the vagina, varying between slight excoriation of the epithelium to deeply invading sores, which, he says, owe their origin to the *mechanical* irritation caused by the contact of morbid secretions with the mucous surfaces, in consequence of which the epithelium, after being kept constantly wet with discharge, is destroyed—we must avoid the too common habit of looking upon all cases of uterine inflammation and lesions as alike, and treating them by fixed rules, without regard to the specific character with which each case may be invested.

For instance, cases sometimes present themselves in which large and extensive uterine ulceration is present, with apparent phagedenic characters, with hardened base and intense pain, evincing most of the constitutional symptoms of carcinoma—wasting, anxiety, languor, and exhaustion on exertion; yet such cases are not necessarily malignant in their nature, and will heal under properly directed treatment.

The most experienced practitioner may be deceived in such a case, and find his treatment result in recovery, in place of death, as he had anticipated. And yet there is usually a *je ne sais quoi* about these cases that leads the practitioner to submit them to treatment almost contrary to his own judgment and expectation of beneficial result.

